# Strengths and weaknesses of the German translation of the Inflexible Eating Questionnaire and of eating disorder assessment in general

**DOI:** 10.3389/fpsyg.2022.1002463

**Published:** 2022-12-19

**Authors:** Anna Schultz, Linda Maurer, Rainer W. Alexandrowicz

**Affiliations:** Department of Methods, Institute of Psychology, University of Klagenfurt, Klagenfurt, Austria

**Keywords:** Inflexible Eating, IEQ, screening, SEM, invariance, Eating Disorder Examination - Questionnaire

## Abstract

**Objective:**

The present article introduces the German translation of the Inflexible Eating Questionnaire (IEQ-G), performs a psychometric evaluation, and explores the relationship of Inflexible Eating to the subscales of the Eating Disorder Examination-Questionnaire (EDE-Q) and Obsessive-Compulsive (OC) symptoms.

**Methods:**

The cross-sectional study was carried out in the German-speaking area. A paper and pencil survey was completed by 612 females and 442 males of the general population.

**Results:**

SEM analyses showed that the IEQ-G allows for calculating a total score and invariance tests were mostly promising. As a side result, the original 4-factorial structure of the EDE-Q could not be replicated, but a 3 dimensional solution proved convincing. From a psychometric point of view, the IEQ-G outperformed the EDE-Q. On a latent level, Inflexible Eating was remarkably strong related to OC-symptoms and the EDE-Q subscales.

**Discussion:**

The detail analyses revealed that Eating Disorder assessment in general lacks subgroup-specific aspects, for instance, regarding gender or dietary preferences, important for early diagnosis and screening of ED. The IEQ-G proved applicable in a German speaking adult population and recommends itself for cross-cultural studies.

## 1. Introduction

Eating Disorders (ED; regarding both full and subthreshold/partial syndrome) affect over 13% of female adolescents (Stice et al., 2013). At the population level, lifetime prevalence of ED ranges between 0.5 and 1%, being 3 to 8 times higher in women compared to men (Hudson et al., 2007; Preti et al., 2009).

One important aspect of ED is Dietary Restraint, which covers all forms of cognitive efforts to restrict caloric intake with the aim of loosing or maintaining weight (Herman and Mack, 1975; Wadden et al., 2002). Characterizing it as either entirely beneficial or harmful seems too short-sighted to conclude, as two dimensions, i.e., rigid and flexible control (Westenhoefer, 1991), with opposite consequences are involved (Westenhoefer et al., 1999, 2013; Stewart et al., 2002; Schaumberg et al., 2016). Rigid control over eating adopts a radical “all or nothing” approach. Periods of strict diet alternate with periods of abundant consumption of food of certain composition (e.g., high in fat and/or sugar). In contrast, the flexible approach abstains from a classification system (“good” vs. “bad” or “allowed” vs. “forbidden” food) and is thus more mobile. Instead of excluding certain foods completely, they are consumed in limited quantities without guilt (Westenhoefer, 1991; Westenhoefer et al., 1999; Duarte et al., 2017; Hagerman et al., 2021). A mismatch between internal (e.g., hunger) and external stimuli (e.g., food odor) as can be found in former dimension is associated with pathological dietary behaviors (Mann and Ward, 2001; Brown et al., 2012; Linardon, 2018) and lower intuitive eating (Tylka and Kroon Van Diest, 2013; Strodl et al., 2020).

Currently, the Eating Disorder Examination-Questionnaire (EDE-Q; Fairburn and Beglin, 1994) addresses a broad range of ED symptoms, such as Diatary Restraint, and is thus widely used to screen for and assess ED. The EDE-Q covers symptom severity in both, clinical and general population studies (Smith et al., 2017; Christian et al., 2020). It builds on the Eating Disorder Examination interview (EDE; Cooper and Fairburn, 1987) forming the ground for clinical diagnoses. The original 28 item EDE-Q self-report splits into the 4 subscales Restraint (RS), Eating Concern (EC), Weight Concern (WC), and Shape Concern (SC; Fairburn and Beglin, 1994). Short versions were 18 item (female) and 16 item (male; Carey et al., 2019), along with a 13 item (Lev-Ari et al., 2021), a 12 item (Gideon et al., 2018), an 8 item (Kliem et al., 2016), and a 7 item (Grilo et al., 2015) version. Their psychometric evaluations (mostly applying Exploratory Factor Analyses) yielded varying results: Peterson et al. (2007), Aardoom et al. (2012), and Friborg et al. (2013) found a 4-factorial solution, yet with items allocated differently to the factors as originally proposed; likewise Peterson et al. (2007), Hilbert et al. (2007), Darcy et al. (2013), White et al. (2014), Grilo et al. (2015), Zohar et al. (2017), Carey et al. (2019), and Heiss et al. (2020) found a 3-factorial solution, in which mostly items from the SC and WC scales formed a common factor next to the RS and EC subscale. This is in line with prominent theories of body image (e.g., self-discrepancy theory, objectification theory; Cash, 2012; Vartanian, 2012). In addition, Penelo et al. (2013) and Rica et al. (2022) reported a 2-factorial structure (RS and EC+WC+SC). Moreover, in some analyses items were dropped from the final solution (see Rand-Giovannetti et al., 2020 Table 1 for an overview), so that no overarching latent structure is discernible for the instrument. Nevertheless, all these versions and flavors of the EDE-Q are used in various studies.

Despite the EDE-Q/RS, several measures capture different aspects of Dietary Restraint (e.g., the Cognitive Restraint subscale of the Three Factor Eating Questionnaire of Stunkard and Messick, 1985 or the Dietary Intent Scale of Stice et al., 2004). However, they only focus on the behavioral aspects of Dietary Restraint (e.g., skipping meals for weight loss or avoiding “bad/forbidden” foods) and disregard the underlying psychological processes, like psychological (in)flexibility. In general, the construct of psychological flexibility is defined as “the ability to contact the present moment more fully as a conscious human being, and to change or persist in behavior when doing so serves valued ends” (Hayes et al., 2006, p. 6). The absence of psychological flexibility is characterized by maladaptive self-rules, avoidance, and suppression (Hayes et al., 2006). According to this construct, such behavioral aspects are the root of different types of psychopathologies including ED (Rawal et al., 2010; Masuda et al., 2011). Thus, Dietary Restraint may become problematic if its coupled with psychological inflexibility (Lillis and Kendra, 2014). Those affected believe that they have to consistently follow a set of self-imposed dieting rules and feel empowered or distressed when these rules are adhered or not adhered to, respectively. Moreover, internal (e.g. hunger and satiety) and external cues (e.g. specific social contexts) are not respected or followed (Duarte et al., 2017). Addressing this gap, Duarte et al. (2017) proposed the concept of Inflexible Eating and developed the Inflexible Eating Questionnaire (IEQ) to capture the said psychological features underlying rigid dietary control. It underwent psychometric analyses, using both, Exploratory Factor Analysis (EFA; Duarte et al., 2017), and Confirmatory Factor Analysis (CFA; Duarte et al., 2017; Linardon et al., 2019; Tie et al., 2022), in addition to path models (Duarte et al., 2016). These analyses were performed for the Portuguese (Duarte et al., 2017), English (Linardon et al., 2019), and Chinese (Tie et al., 2022) versions.

### 1.1. Further aspects associated with ED and disordered eating behavior

Evidence indicates that ED and disordered eating behavior are associated with Obsessive-Compulsive Disorder (OCD; Altman and Shankman, 2009). Previous studies showed that both pathologies are characterized by an intense preoccupation with a particular stimulus; food or weight/shape in ED and, for instance, contamination in OCD. Such stimuli elicit negative affects followed by compensatory behavior (e.g., purging in ED or washing in OCD) to reduce the negative affect (Altman and Shankman, 2009). Given their somewhat great similarity on the functional level, high comorbidity rates of ED and OCD are not surprising. Although the increased prevalence of OCD in ED compared to the general population is an established finding (Kaye et al., 2004; Ulfvebrand et al., 2015), prevalence rates vary highly. According to Kaye et al. (2004) approximately 41% of individuals with an ED diagnosis have a lifetime OCD comorbidity. Swinbourne et al. (2012), on the other hand, found that 5% of women presenting for treatment of an ED met criteria for OCD. Mandelli et al. (2020) summarize that prevalence rates range between 3 and 53% in ED populations. Comorbidity with OCD is associated with worse ED outcome (Wentz et al., 2009; Carrot et al., 2017) and greater risk of relapse (Berends et al., 2018). Simpson et al. (2013) report, the effectiveness of treating OCD and ED simultaneously, so that, at discharge, both patients OCD severity and ED symptoms reduced significantly.

Regarding gender, ED are historically conceptualized as a problem of young females. Thus, classification systems of ED are based on female representations and, in turn, assessment methods are developed with this premise in mind (Mitchison and Mond, 2015). However, as shown by Murray et al. (2017), ED have been reported equally in men and women since the beginning. While only 1% of peer-reviewed manuscripts deal with male representations of ED (Murray et al., 2016), there is consensus that male and female ED differ somewhat in terms of risk factors, clinical presentation, comorbidity, and outcomes (Mitchison et al., 2013; Raevuori et al., 2014; Murray et al., 2017). In reviewing recent studies, Murray et al. (2017) highlight differences in symptom presentation between the sexes. Whereas the nature of Dietary Restraint in female Anorexia Nervosa (AN) is oriented toward thinness and emaciation, males may thrive for a lean and muscular appearance (Pope et al., 2000; Yanover and Thompson, 2010). Consistent with this so-called “Adonis-Complex,” males suffering from AN reported to be less concerned about weight while being equally concerned (compared to females) about shape (Muise et al., 2003; Strober et al., 2006). This overvaluation of shape especially in males is also present in muscle dismorphia (see Murray et al., 2012). Higher hospital admission and desired BMI in male AN patients (Gueguen et al., 2012) might further reflect these different shape ideals (i.e., masculine, with large shoulders and narrow hips/waist; Murray et al., 2017). Likewise, male Bulimia Nervosa (BN) may present itself somewhat different to female BN. While both sexes report eating large portions, males seem less likely to lose control (Lewinsohn et al., 2002; Striegel-Moore et al., 2009) or worry about their eating behavior during binge episodes (Lavender et al., 2010). Although the clinical significance of “cheat meals” and “cheat days” to male presentation of BN remains unclear (Murray et al., 2017), the “large” amount of food consumed (up to 9000 calories) and the reported loss of control while eating may resemble objective binge episodes (Pila et al., 2017). This dietary phenomenon seems to emerge among body builders (Goldfield et al., 2006; Chaba et al., 2019) and non-body builders (Pila et al., 2017) as well. “Cheat” or binge episodes are followed by compensatory measures, for instance, excessive exercise and a more rigid adherence to their nutritional plan (i.e., Dietary Restraint; Connan, 1998). Hence, both sexes appear to be equally impaired by binge eating (Striegel et al., 2012; Gilmartin et al., 2022). Regarding compensatory behaviors, males appear more likely to display non-purging behaviors, like extreme dietary restriction and excessive exercise (Lavender et al., 2010) while females appear more likely to display “typical” purging behavior, like laxative use (Striegel-Moore et al., 2009).

### 1.2. Research question

In general, ED feature a vast number of adverse consequences, for instance, cardiovascular complications (Casiero and Frishman, 2006), gastrointestinal disturbances (Zipfel et al., 2006), dental problems (Mehler, 2011), non-suicidal self-injury (Cucchi et al., 2016), or high mortality rates (Smink et al., 2012). Therefore, early detection is key. The IEQ may be used as a screening tool, as it measures the rigid adherence to eating rules, which, according to Duarte et al. (2016), play an important role in the progression of disordered eating behaviors to clinically relevant cases.

As there is no German translation of the IEQ available yet (though German being the 2nd most spoken language in Europe; Bohn, 2018), the present study sets out to compile such a version and to explore its psychometric properties. Furthermore, the study is devoted to further inspect the latent structure and the interplay of the IEQ-G, EDE-Q and OC subscale of the SCL-90-R in various subgroups.

## 2. Materials and methods

### 2.1. Design

The cross-sectional study was carried out in the German-speaking area (i.e., Germany and Austria) to a) assess the psychometric properties of the translated German version of the IEQ and b) investigate latent correlations between the IEQ-G and other measures related to eating psychopathology.

### 2.2. Participants and sampling

Paper and pencil data were collected in March 2020. Participants were recruited by means of a convenience sample involving a snowball approach starting with Psychology students. Each student filled out the questionnaire him- or herself and distributed 10 further exemplars to respondents of varying gender and age. All participants were fully informed about the aims of the study and about the confidentiality of the data, and they were also assured that the data would be used only for the purpose of the research. Informed consent was obtained from each participant before participating in the study. Every precaution was taken to protect the privacy of research subjects and the confidentiality of their personal information. Overall, 1,218 participants completed the forms.

### 2.3. Measures

The paper-pencil questionnaire included a section dealing with background information (e.g., gender, age, eating preferences), the EDE-Q, the OC subscale of the SCL-90-R, and the IEQ-G.

#### 2.3.1. Eating Disorder Examination - Questionnaire (28 items)

The EDE-Q (Fairburn and Beglin, 1994; German version by Hilbert and Tuschen-Caffier, 2006) consists of 28 items addressing key features of ED psychopathology within the last 28 days. Twenty-two of these items form the following subscales: Restraint (5 items), Eating Concern (5 items), Weight Concern (5 items) and Shape Concern (8 items). The remaining 6 items represent diagnostically relevant core behaviors, such as laxative abuse. Participants are asked to rate each item according to the frequency (“0 = never” to “6 = every day”) of the said behavior or the severity of symptoms (“0 = not at all” to “6 = significantly”). For the German version, Cronbach's α ranged from 0.85 (WC) to 0.93 (SC) in a combined sample (samples with AN, BN, atypical ED, and nonclinical, subclinical, and psychiatric comparison groups). As the 4-factorial structure could not be established, the subscales WC and SC were combined. Cronbach's α for the WS/SC subscale was 0.95 in the combined sample (Hilbert et al., 2007). Internal consistency (ω) for a 2-factorial structure for the Spanish version was between 0.80 for RS and 0.92 for EC/WC/SC, and 0.94 for the Global score (Penelo et al., 2013). Additionally, Penelo et al. (2013) reports a satisfactory 2-week test-retest reliability (intra-class correlation coefficients ≥ 0.84; Cohen's Kappa ≥ 0.56), and evidence for convergent validity with external measures.

#### 2.3.2. Symptom Checklist 90-R/Obsessive-Compulsive subscale

The SCL-90-R (Derogatis, 1977; German version by Franke, 2002) is a 90 item self-administered questionnaire measuring the subjective severity psychopathological symptoms. Participants rate each item on a 5-point Likert-type scale ranging from “not at all” (0) to “extremely” (4). One of the 9 subscales, the Obsessive-Compulsive (OC), was included in the current study. Cronbach's α for the OC subscale ranged between 0.86, 0.85, and 0.75 in a psychosomatic outpatient, a primary care, and a reference sample, respectively (Schmitz et al., 2000).

#### 2.3.3. Inflexible Eating Questionnaire

The IEQ (Duarte et al., 2017) records the rigid adherence to self-imposed eating rules. Likewise, the instrument maps the tendency to feel encouraged or distressed when such rules are followed or violated, respectively. The items are the result of both an extensive literature review on the role of dietary restrictions and eating rules in ED and clinical experience with ED and obesity. Participants respond to the 11 items using a five-point rating scale (“1 = strongly disagree” to “5 = strongly agree”). Analyses using EFA and CFA suggested a unidimensional structure of the IEQ. In addition, Duarte et al. (2017) found evidence of internal consistency (α_*CR*_ = 0.90), temporal stability (4-week retest reliability = 0.84), and convergent validity (AVE = 0.77).

### 2.4. Translation of the IEQ

In this study, we combined a committee and back-translation approach (Brislin, 1970) to compile the German version of the IEQ and ensure its semantic equivalence. The research team consisting of three bilinguals translated the English version of the IEQ to German (IEQ-G). Subsequently, a professional translator and native speaker (BE) with excellent command of German performed a blinded back-translation which the research team reviewed and discussed. Discrepancies between the back-translation and the English version were discussed and the German items were adjusted where necessary. Specifically, Items 2, 6, 8, and 9 required minor modifications. For instance, the first version of Item 2 read “Wenn ich eine meiner Essensregeln nicht einhalten kann, dann versuche ich das durch noch strengere Einhaltung dieser Regeln auszugleichen.” Here, the “kann” was eliminated as it represented a different mode. In German, the verb “können” suggests the possibility, but not the occurrence of such a situation. In addition, an attempt was made to eliminate ambiguous meanings in the item wordings. Therefore, we replaced “dieser Regeln” in Item 2 with “meiner Regeln” because the IEQ does not target arbitrary but self-imposed eating rules. Likewise, we adjusted Item 8 reading “Selbst wenn ich zufrieden mit meinem Gewicht bin, lasse ich keine Lockerung meiner Essensregeln zu.”: By substituting the verb “erlauben” with “lassen,” we emphasized on inflexible behavior that does not allow any exception.

In the course of this revision, we attempted to generate “naturally sounding” items, which, on the one hand are not bound to the grammatical structures of the English language but are nevertheless faithful to the English version (i.e., semantic equivalence; Flaherty et al., 1988). The initial translation of Item 6 was “Bei jeder (auch nur geringen) Veränderung meines Körpergewichts achte ich ganz besonders auf die Einhaltung meiner Essensregeln.”. After reaching consensus, it was replaced by “wird das Einhalten meiner Essensregeln zur Priorität.” “Etwas zur Priorität machen” seemed to be a more common phrase in German-speaking countries. In the same vein, Item 9 (initially reading “Es macht mich stolz, wenn ich meine Essensregeln streng einhalten kann.”) was rephrased: “genaue Einhalten von Regeln” became “strengem Einhalten von Regeln.”

This pre-final version of the IEQ-G was then sent again to a professional translator who carried out a new back-translation. The research team detected no further discrepancies between the back-translated and the original IEQ items. Subsequently, this version was tested in a pilot study. The original version of the IEQ was presented to 40 native English speakers (living in England, America, and Australia), to assess the adequacy of the translation. Their data was then matched according to gender, age, dietary preference (omnivorous diet vs. vegetarian diet vs. vegan diet vs. other preferences) and body-self perception (Feel; (rather) too thin vs. comfortable vs. (rather) too fat) with data from a second pilot which presented the IEQ-G. Overall, participants' response patterns indicated sufficient agreement.

### 2.5. Data analysis

First, we investigated the factorial structure of the EDE-Q, SCL-90-R/OC, and the IEQ-G using CFA. All analyses were performed using maximum likelihood estimation with robust standard errors (MLR) suitable for nonnormal data (Savalei and Rosseel, 2022). For the EDE-Q, we compared three models: (a) The originally proposed 4-factor model (RS, EC, WC, and SC), (b) A 3-factor model collapsing WC and SC while retaining RS and EC, (c) A 2-factor model that retains RS but collapses EC, WC, and SC.

The fit of each CFA model was evaluated based on the Satorra-Bentler scaled χ^2^-test (Satorra and Bentler, 1994), the normed χ^2^ (NC; Tabachnick et al., 2007), the Comparative Fit Index (CFI), Tucker Lewis Index (TLI), the Root Mean Square Error of Approximation (RMSEA), and the Standardized Root Mean Square Residual (SRMR). Model fit is considered “excellent” if CFI and TLI ≥0.95, RMSEA and SRMR ≤ 0.05 and “adequate” if CFI and TLI range from 0.90 to 0.94 and RMSEA and SRMR range ≤ 0.06 (Hu and Bentler, 1999). Another criterion for adequate fit was NC between 2 (Tabachnick et al., 2007) and 5 (Schumacker and Lomax, 2004). Robust McDonald's omega coefficients were used to assess reliability while accounting for the nonnormality of data (Zhang and Yuan, 2020).

Furthermore, we investigated the invariance (cf. Byrne, 2008; Hirschfeld and Von Brachel, 2014) of the IEQ with respect to Gender (female vs. male), Age ( ≤ 34 vs. ≥35), BMI groups (underweight vs. normal weight vs. overweight), Diet groups (omnivorous diet vs. other preferences), and body-self perception (Feel; (rather) too thin vs. comfortable vs. (rather) too fat). As for the BMI, the self-reported height and weight in the EDE-Q were converted to the Quetelet's index of body mass (kg/m^2^). Subsequently, BMI scores were classified into underweight (< 18.50 kg/m^2^), normal weight (18.50–24.99 kg/m^2^), and overweight (≥ 25.00 kg/m^2^) based on the classification proposed by the WHO (World Health Organization, 2000).

We first estimated a baseline model for each grouping variable with loadings being freely estimated (= configural invariance). Next, loadings and intercepts for each group were constrained, respectively, to examine factorial (metric) and strong factorial (scalar) invariance. Finally, residual (strict) and mean (structural) invariance were tested by constraining the means of the latent variables and residual variances of the observed variables, respectively. Consecutive models were tested using the χ^2^-test.

Additionally, the best fitting measurement models of all questionnaires were entered simultaneously in a correlated factor model to estimate latent correlations (ϕ) between all constructs. Again, a maximum likelihood estimation with robust standard errors (MLR) was used to account for the nonnormal data.

Statistical analyses were performed using R (R Core Team, 2019; version 3.6.1); the semTools (Jorgensen et al., 2020; version 0.5–3) and the lavaan packages (Rosseel, 2012; version 0.6-6) were used for the CFA and SEM. The coefficientalpha (Zhang and Yuan, 2020; version 0.7) package was used to compute robust alpha or omega coefficients and the tau equivalence and homogeneity *F*-tests. The online tool developed by Carter and Colwell (2013) was used for the Satorra-Bentler scaled χ^2^ difference testing (Δχ^2^; Satorra and Bentler, 2010) of consecutive models (i.e., the three examined EDE-Q models and invariance testing). The significance level was set to α = 0.05.

## 3. Results

### 3.1. Sample characteristics

From 1218 respondents 1073 were suitable for analysis (117 input errors; 28 not scoring in any of the ED inventories). There were no suspicious missing patterns and 90.39% had no missings at all. Table 1 lists the sample characteristics and the online Supplement details the items' distributions (Supplementary Table S1). The item responses do not follow a normal distribution. Several items (EDE-Q-19, EDE-Q-21, and SCL-R/OC-10) even exceed a skewness of +3 and kurtosis ranges from –1.25 (IEQ-09) to 12.85 (EDEQ-19).

**Table 1:** Characteristics of study participants.

		***n***	**%**
Total		1,073	100.0
Gender	Male	442	41.2
	Female	612	57.0
	Divers	19	1.8
Age^*a*^	≤ 19	119	11.1
	20–24	382	35.6
	25–29	171	15.9
	30–34	53	4.9
	35–39	31	2.9
	40–44	40	3.7
	45–49	60	5.6
	≥50	217	20.2
Diet	Omnivorous	749	69.8
	Vegetarian	195	18.2
	Vegan	58	5.4
	Paleo	10	0.9
	Raw food	5	0.5
	Keto	11	1.0
	Other	45	4.2
BMI	< 18.50	58	5.4
	18.50–24.99	695	64.8
	≥ 25.00	320	29.8
Feel	(Rather) too thin	83	7.7
	Comfortable	624	58.2
	(Rather) too fat	366	34.1

^*a*^Age was collected in categories to support anonymity.

### 3.2. Confirmatory factor analyses

#### 3.2.1. Eating Disorder Examination - Questionnaire

CFA using maximum likelihood estimation was carried out to investigate the factor structure of the EDE-Q. Following the inconclusive results of previous studies, we inspected a 2-, 3-, and 4-factorial model. Table 2 displays the comparison of these three models. Note that the original version of the EDE-Q scored item 8 on both the WC and the SC subscale. This within-item-multidimensionality caused estimation problems in our analyses (not positive definite covariance matrix), so that we assigned this item to WC only (the same problem has been reported Rica et al., 2022, for example). Even assigning item 8 only to the WC or the SC subscale (because the item addresses both weight and shape concerns), respectively, still yielded a not positive definite covariance matrix. Thus, the model was not deemed to be acceptable (Table 2 shows the results with item 8 assigned to WC; the results of item 8 assigned to SC were virtually identical).

**Table 2:** Fit index values for the tested models (*n* = 1,073).

	**EDE-Q**		
	**4-factor solution^*b*^**	**3-factor solution**	**2-factor solution**
	**RS/EC/WC/SC**	**RS/EC/WC+SC**	**RS/EC+WC+SC**
χ^2^	2,229.307	2,268.497	2,452.286
*df*	203	206	208
NC	10.982	11.012	11.790
SF	1.648	1.656	1.675
Δχ^2^	–	37.652	96.627
Δ*df*	–	3	2
NC_Δ_	–	12.551	48.314
SF_Δ_	–	2.197	3.632
Δ_*p*_	–	< 0.001	< 0.001
CFI^*a*^	0.790	0.785	0.763
TLI^*a*^	0.761	0.759	0.737
RMSEA^*a*^	0.124	0.124	0.130
SRMR^*a*^	0.076	0.077	0.078

χ^2^ = Satorra-Bentler scaled chi-square; NC = normed chi-square (χ^2^/*df*); SF = scaling factor of the Satorra-Bentler scaled chi-square statistics; Δχ^2^ = Satorra-Bentler scaled chi-square difference test; Δ*df* = differences in degrees of freedom; NC_Δ_ = NC of difference test; SF_Δ_ = difference test scaling factor.

^*a*^Robust fit indices.

^*b*^Due to estimation problems, this model will not be considered further (see text).

**Figure 1:**
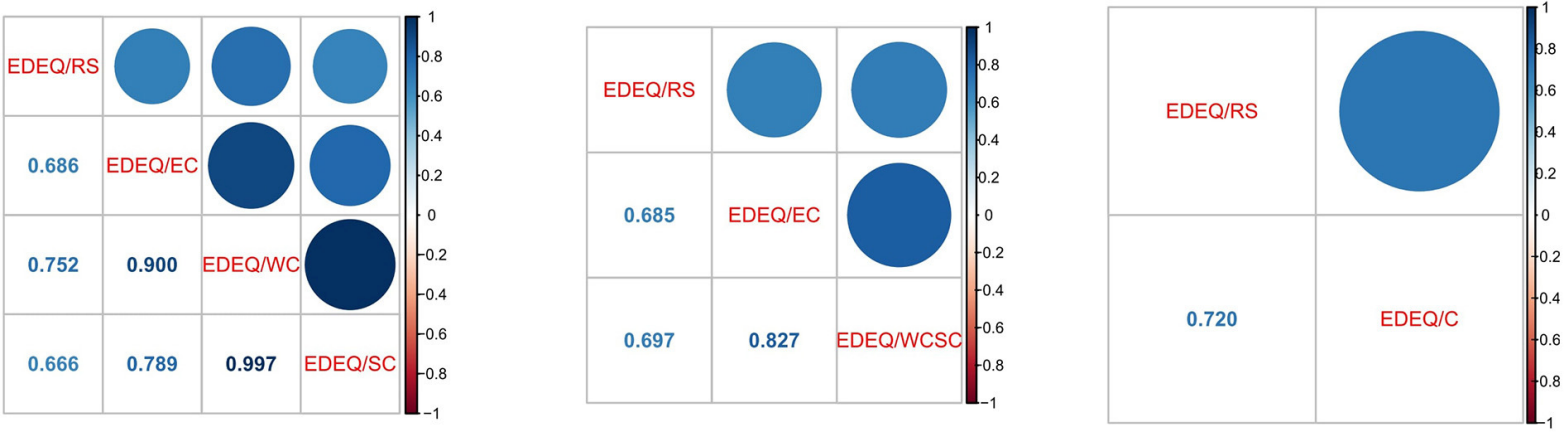
Latent correlation coefficients of the 4-factorial solution **(left)**, the 3-factorial solution **(middle)**, and the 2-factorial solution **(right)** of the EDE-Q.

Figure 1 displays the latent correlations of all subscales of the EDE-Q of the three examined models.

The high latent correlation of the EDE-Q subscales WC and SC of ϕ = 0.997 indicate almost perfect agreement of these two subscales (Figure 1, left). Therefore, we consider the WC/SC subscales essentially unidimensional and decided to continue our analyses with the 3-factorial solution (Figure 1, middle). Table 3 lists the loadings and fit measures of the 3-factorial solution in greater detail (see the Supplementary Table S2 for the 2- and 4-factorial solution, respectively).

**Table 3:** Standardized loadings and robust fit statistics of the three CFA models estimated separately.

	**EDE-Q (3-factors)**									**SCL-90-R/OC**			**IEQ-G**	
	**Restraint**			**Eating Concern**			**Weight/Shape Concern**							
	**Item**	λ^*^		**Item**	λ^*^		**Item**	λ^*^		**Item**	λ^*^		**Item**	λ^*^
	RS-01	0.824^*^		EC-07	0.641^*^		WCSC-08	0.613^*^		OC-01	0.642^*^		IEQ-01	0.761^*^
	RS-02	0.565^*^		EC-09	0.734^*^		WCSC-12	0.812^*^		OC-02	0.612^*^		IEQ-02	0.766^*^
	RS-03	0.735^*^		EC-19	0.540^*^		WCSC-22	0.705^*^		OC-03	0.665^*^		IEQ-03	0.549^*^
	RS-04	0.765^*^		EC-20	0.798^*^		WCSC-24	0.481^*^		OC-04	0.652^*^		IEQ-04	0.735^*^
	RS-05	0.586^*^		EC-21	0.691^*^		WCSC-25	0.808^*^		OC-05	0.530^*^		IEQ-05	0.583^*^
							WCSC-06	0.581^*^		OC-06	0.552^*^		IEQ-06	0.657^*^
							WCSC-10	0.750^*^		OC-07	0.666^*^		IEQ-07	0.761^*^
							WCSC-11	0.825^*^		OC-08	0.599^*^		IEQ-08	0.624^*^
							WCSC-23	0.685^*^		OC-09	0.687^*^		IEQ-09	0.746^*^
							WCSC-26	0.826^*^		OC-10	0.459^*^		IEQ-10	0.703^*^
							WCSC-27	0.843^*^					IEQ-11	0.777^*^
							WCSC-28	0.807^*^						
**Fit**	**EDE-Q (3-factors)**									**SCL-90-R/OC**			**IEQ-G**	
χ^2^ (*df*)	2, 268.497 (206)^*^									280.711 (35)^*^			309.163 (44)^*^	
NC	11.012									8.020			7.026	
CFI^*a*^	0.785									0.877			0.942	
TLI^*a*^	0.759									0.842			0.928	
RMSEA^*a*^	0.124									0.105			0.084CI 90%>CI 90%	
														
	[0.120-0.129]									[0.094-0.117]			[0.075-0.093]	
SRMR^*a*^	0.077									0.061			0.037	

λ^*^ = standardized loadings;

^*^p < 0.05; χ^2^ = Satorra-Bentler scaled chi-square; NC = normed chi-square (χ^2^/*df*).

^*a*^Robust fit indices.

As shown in Table 3, the NC of 11.012 exceeds the recommended range of 2–5. The standardized factor loadings for the 3-dimensional model ranged from λ^*^ = 0.565 (item 2) to λ^*^ = 0.824 (item 1) for the RS, from λ^*^ = 0.540 (item 19) to λ^*^ = 0.798 (item 20) for the EC, and from λ^*^ = 0.481 (item 24) to λ^*^ = 0.843 (item 27) for the combined Weight and Shape Concern subscale (WC/SC). Overall, fit was poor (robust CFI = 0.785, robust RMSEA = 0.124). Because the tau equivalence test failed [ RS:*F*_(9, 1, 064)_ = 10.75;*p* < 0.001; EC: *F*_(65, 1, 008)_ = 5.968;*p* < 0.001; WC/SC: *F*_(135, 938)_ = 3.047;*p* < 0.001], robust McDonald's Omega (including *F*-test) for the RS, EC, and SC/WC subscale of the EDE-Q were applied [RS: ω = 0.857, *SE* = 0.009; *F*_(5, 1, 068)_ = 5.763;*p* < 0.001; EC: ω = 0.714, *SE* = 0.024; *F*_(5, 1, 068)_ = 3.132;*p* < 0.010 WC/SC: ω = 0.937, *SE* = 0.005; *F*_(54, 1, 019)_ = 4.615;*p* < 0.001].

#### 3.2.2. Symptom Checklist-90-R/Obsessive-Compulsive

CFA using maximum likelihood estimation was carried out to investigate the factor structure of the SCL-90-R/OC. As shown in Table 3, the standardized factor loadings ranged from λ^*^ = 0.459 (item 10) to λ^*^ = 0.687 (item 9). Overall, fit was poor (NC = 8.020; robust CFI = 0.877, robust RMSEA = 0.105). Again, the tau equivalence test failed [*F*_(44, 1, 029)_ = 4.659;*p* < 0.001]. McDonald's omega (robust) for the SCL-90-R/OC was 0.844 [*SE* = 0.008; *F*_(35, 1, 038)_ = 2.989;*p* < 0.001]

#### 3.2.3. Inflexible Eating Questionnaire-German

CFA using maximum likelihood estimation was carried out to investigate the factor structure of the IEQ-G. As shown in Table 3, the standardized factor loadings ranged from λ^*^ = 0.549 (item 3) to λ^*^ = 0.777 (item 11). Overall, fit was quite poor (NC = 7.026; robust CFI = 0.942, robust RMSEA = 0.084). As the tau equivalence test failed [*F*_(54, 1, 019)_ = 7.750;*p* < 0.001], McDonald's omega (robust) for the IEQ-G was applied [ω = 0.919, *SE* = 0.004; *F*_(44, 1, 029)_ = 5.106;*p* < 0.001].

### 3.3. Invariance of the IEQ-G

Next, we examined the measurement invariance of the one-factor model of the IEQ-G 1) between male and female subgroups, 2) between younger ( ≤ 34) and older (≥35) participants, 3) between under-, normal- and overweight subgroups, 4) between omnivorous and subgroups with eating preferences (e.g., vegan diet), and 5) between subgroups who feel too thin, comfortable or too fat, applying the multi-sample procedure. As described in Table 4 configural, metric, scalar, mean, and residual invariance was tested for each subgroup.

**Table 4:** Robust fit indices and Satorra-Bentler scaled χ^2^ differences for multi-sample analyses.

		**χ^2^ (*df*)**	**SF**	**NC**	***Δχ* ^2^**	***Δdf***	**NC_Δ_**	**SF_Δ_**	***p*_Δ_**	**CFI^*a*^**	**TLI^*a*^**	**RMSEA^*a*^**	**SRMR^*a*^**
Gender^*b*^	Configural	342.235 (88)	1.251	3.889						0.944	0.930	0.083	0.036
	Metric	378.251 (98)	1.216	3.860	35.041	10	3.504	0.908	< 0.001	0.940	0.933	0.081	0.051
	Scalar	426.293 (108)	1.196	3.947	49.893	10	4.989	1.000	< 0.001	0.933	0.932	0.082	0.055
	Mean	433.521 (109)	1.194	3.977	7.953	1	7.953	0.978	= 0.005	0.932	0.931	0.082	0.060
	Residual	450.992 (120)	1.189	3.758	16.328	11	1.484	1.140	= 0.129	0.931	0.936	0.079	0.062
Age	Configural	364.495 (88)	1.270	4.142						0.940	0.925	0.086	0.037
	Metric	386.008 (98)	1.229	3.939	13.240	10	1.324	0.868	= 0.211	0.940	0.932	0.082	0.042
	Scalar	438.414 (108)	1.207	4.059	55.237	10	5.523	0.991	< 0.001	0.932	0.931	0.083	0.046
	Mean	439.675 (109)	1.206	4.034	0.986	1	0.986	1.098	= 0.321	0.932	0.931	0.083	0.046
	Residual	450.929 (120)	1.200	3.758	9.528	11	0.866	1.141	= 0.573	0.932	0.938	0.079	0.046
BMI	Configural	459.645 (132)	1.225	3.482						0.932	0.914	0.092	0.039
	Metric	493.328 (152)	1.189	3.246	24.702	20	1.235	0.951	= 0.213	0.931	0.925	0.086	0.046
	Scalar	533.722 (172)	1.167	3.103	36.294	20	1.815	1.000	= 0.014	0.928	0.931	0.083	0.048
	Mean	539.208 (174)	1.165	3.099	5.361	2	2.681	0.993	= 0.069	0.927	0.931	0.083	0.052
	Residual	568.410 (196)	1.164	2.900	28.935	22	1.315	1.156	= 0.147	0.926	0.938	0.079	0.053
Diet	Configural	369.835 (88)	1.229	4.203						0.938	0.923	0.086	0.038
	Metric	404.393 (98)	1.190	4.126	31.531	10	3.153	0.847	< 0.001	0.935	0.927	0.083	0.049
	Scalar	460.481 (108)	1.175	4.264	58.207	10	5.821	1.028	< 0.001	0.926	0.925	0.085	0.053
	Mean	503.256 (109)	1.171	4.617	65.288	1	65.288	0.739	< 0.001	0.918	0.917	0.089	0.080
	Residual	546.540 (120)	1.160	4.555	42.506	11	3.864	1.051	< 0.001	0.912	0.919	0.088	0.084
Feel	Configural	416.995 (132)	1.250	3.159						0.935	0.919	0.087	0.040
	Metric	461.352 (152)	1.201	3.035	37.420	20	1.871	0.878	= 0.010	0.932	0.927	0.083	0.054
	Scalar	511.034 (172)	1.178	2.971	47.762	20	2.388	1.003	< 0.001	0.927	0.937	0.081	0.058
	Mean	577.626 (174)	1.176	3.320	76.982	2	38.491	1.004	< 0.001	0.914	0.918	0.087	0.100
	Residual	674.417 (196)	1.170	3.441	97.795	22	4.445	1.123	< 0.001	0.898	0.914	0.089	0.107

χ^2^ = Satorra-Bentler scaled chi-square; SF = scaling factor of the Satorra-Bentler scaled chi-square statistics; NC = normed chi-square (χ^2^/*df*); Δχ^2^ = Satorra-Bentler scaled chi-square difference test; Δ*df* = differences in degrees of freedom; NC_Δ_ = NC of difference test; SF_Δ_ = difference test scaling factor.

^*a*^Robust fit indices.

^*b*^19 (1.8%) observations, who identified themselves as “Divers”, were omitted from gender split (*n* = 1, 054).

In Table 4, we find significant χ^2^-statistics for all models. Moreover, the χ^2^ difference tests indicate metric invariance to hold for the splits Age and BMI. Note that for BMI, only the step from metric to scalar invariance yielded a significant difference test (*p* = 0.014), while all other restrictions did not. Considering NC_Δ_ and the robust fit statistics, metric invariance held for all models. Furthermore, scalar invariance was found for Gender and Feel splits and residual for BMI split. Generally, the robust fit measures CFI, TLI and SRMR were quite acceptable for most of the splits and decreased only slightly with the various restrictions regarding parameter invariance.

### 3.4. Latent associations between instruments

**Figure 2:**
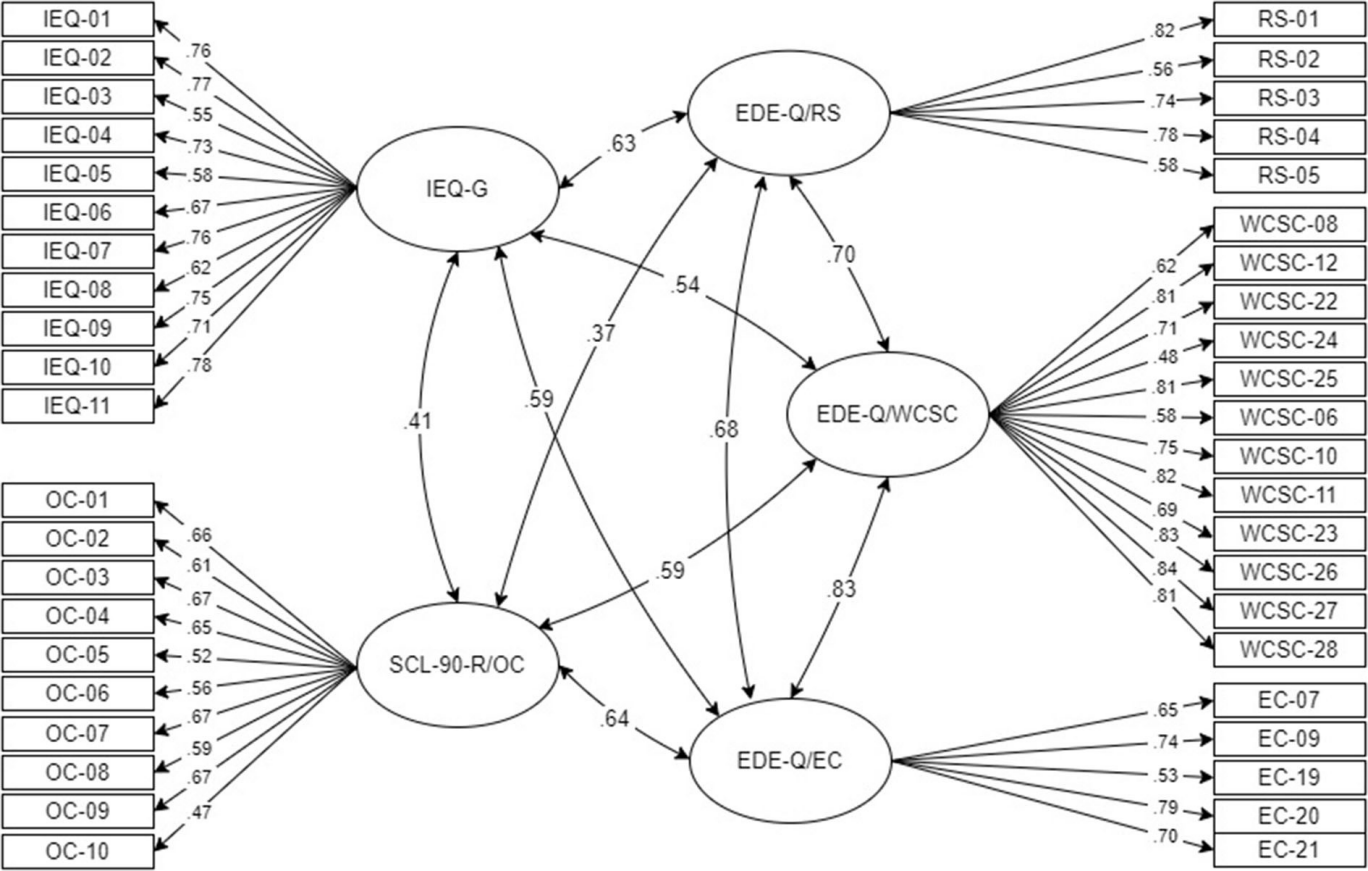
Depiction of SEM with standardized loadings and latent correlation coefficients.

A SEM was applied in conjunction with the CFAs in Section 3.2 to examine the relationships between all latent constructs. Figure 2 depicts the standardized loadings and latent correlations between all constructs. For the IEQ-G, standardized factor loadings ranged from λ^*^ = 0.55 (item 3) to λ^*^ = 0.78 (item 11), for the SCL-90-R/OC from λ^*^ = 0.47 (item 10) to λ^*^ = 0.67 (item 3, 7, and 9), for the EDE-Q/RS between λ^*^ = 0.56 (item 2) to λ^*^ = 0.82 (item 1), for the EDE-Q/WCSC from λ^*^ = 0.48 (item 24) to λ^*^ = 0.84 (item 27), and for the EDE-Q/EC from λ^*^ = 0.53 (item 19) to λ^*^ = 0.79 (item 20). The strongest latent correlation was found between the IEQ-G and EDE-Q/RS (ϕ = 0.63). The latent correlation between IEQ-G and EDE-Q/EC, EDE-Q/WCSC, and SCL-90-R/OC were ϕ = 0.59, ϕ = 0.54, and ϕ = 0.41, respectively. The internal structure of the EDE-Q is comparable to that of the EDE-Q model in Section 3.2.1 (Figure 1). Fit indices revealed almost moderate fit [χ^2^(*df*) = 4, 452.848(850), NC = 5.239, robust CFI = 0.816, robust TLI = 0.804, robust RMSEA = 0.074, robust SRMR = 0.064].

Table 5 shows the correlations among the latent constructs for the various subgroups.

**Table 5:** Latent correlations (ϕ) per split group.

		**Group**	***n***	**SCL-90-R/OC**	**EDE-Q/RS**	**EDE-Q/EC**	**EDE-Q/WCSC**
IEQ-G		**Global**		**0.412**	**0.631**	**0.589**	**0.539**
		Female	612	0.445	0.659	0.662	0.582
	Gender^*a*^	Male	442	0.335	0.570	0.436	0.431
	Age	≤ 34	725	0.414	0.655	0.595	0.563
		< 18.50	58	0.755	0.686	0.729	0.622
		18.50 − 24.99	695	0.407	0.597	0.592	0.561
		Comfortable	624	0.287	0.543	0.493	0.411
	Feel	(Rather) too fat	366	0.390	0.656	0.635	0.561
SCL-90-R/OC		**Global**			**0.369**	**0.638**	**0.587**
		Female			0.427	0.675	0.651
		18.50 − 24.99			0.414	0.634	0.626
	Diet	Omnivorous			0.311	0.589	0.573
		(Rather) too thin			.528	0.605	0.617
		Comfortable			0.219	0.610	0.461
	Feel	(Rather) too fat			0.341	0.625	0.606
EDE-Q/RS		**Global**				**0.684**	**0.697**
		Female				0.748	0.723
	Age	≤ 34				0.747	0.749
		< 18.50				.937	.885
		18.50 − 24.99				0.683	.738
	Diet	Omnivorous				0.639	0.685
		(Rather) too thin				.911	.863
		Comfortable				.518	.583
	Feel	(Rather) too fat				.661	.605
EDE-Q/EC		**Global**					**0.826**
		Female					.841
	Age	≤ 34					0.830
		< 18.50					.940
		18.50 − 24.99					.813
	Diet	Omnivorous					0.824
		(Rather) too thin					0.974
		Comfortable					0.785
	Feel	(Rather) too fat					0.787

For better comparability, the global correlations of Figure 2 are repeated at the top of each block and printed in bold font.

^*a*^19 (1.8%) observations, who identified themselves as “Divers,” were omitted from gender split (*n* = 1, 054).

For all scales, latent correlations in females, underweight, and the subgroup with dietary preferences were higher compared to the global correlation pattern. In comparison, men, people who feel comfortable, and people without any dietary preferences (omnivore) show lower correlation patterns compared to the global one. When looking at the correlation between the IEQ-G and the other scales, the younger ( ≤ 34) subgroup shows slightly higher correlation coefficients compared to the global ones, in contrast to the older subgroup (≥35), whose correlation coefficients were lower compared to the global ones.

## 4. Discussion

The aim of the present study was to examine the psychometric properties and invariance of the IEQ-G in a large sample of German speaking adults. Overall, our results showed that the IEQ-G has quite acceptable psychometric properties, excelling those of the EDE-Q. All χ^2^-tests of global fit yielded significant results, but this may partially due to the large sample of more than 1,000 observations (Satorra and Bentler, 1994).

Regarding invariance, most of the χ^2^-tests were significant [Table 4, Column 1, χ^2^(*df*)], so that, from a statistical point of view, the invariance property has to be rejected. However, considering the large sample, the NC and NC_Δ_ statistics (Column 3 and 6), and from a substantive perspective, all splits would render at least metric and for Gender and Feel also scalar invariance. The BMI split even attained full invariance according to the NC_Δ_. This could be elaborated in further studies to ascertain, whether group specific norms are required for practical assessment. Gender invariance of the IEQ-G could not be fully established, although the NC_Δ_ statistics also support metric or even scalar invariance. This is in line with Tie et al. (2022), who also reported metric invariance for the C-IEQ (adolescent version) in a sample of Chinese high school students. Interestingly, the BMI split of the invariance tests of the IEQ-G were (except for one, viz. metric/scalar) not significant, whereas the “subjective” point of view (I feel (rather) too thin/comfortable/(rather) too fat) yielded significant results for all restrictions and was only acceptable with respect to configural invariance according to the χ^2^ difference tests. This shows that the “objective” BMI is from an assessment perspective of much lesser importance than how the respondent considers him- or herself (Messer and Linardon, 2021). Hence, the latter should gain more focus/attention in screenings than the BMI—the more, as assessing the BMI is known to be difficult to ask for. Not only may respondents alter their size and/or weight in questionnaires (Brener et al., 2003; Engstrom et al., 2003), it seems that gender differences are present in these alterations. According to Park (2011), girls were more prone to overestimate their weight while boys were more likely to underestimate it.

Altogether, it seems save to conclude that the IEQ-G has acceptable psychometric properties, which is in line with Duarte et al. (2017) and Linardon et al. (2019). The unidimensional structure allows for easily calculating a global score, thus fostering its application in screening studies. In the Practice Guidelines for the Treatment of Patients with ED, the American Psychiatric Association (2006) notes that both early detection of ED and intervention may prevent chronification. In the same line, Herzog et al. (1999) and the American Psychiatric Association (2006) postulate that identifying ED related problems is necessary before they become intractable. In addition, Fitzsimmons-Craft et al. (2019) highlight a treatment gap, particularly among adolescents, between those who need therapy/therapeutic interventions and those who receive them. Considering that Inflexible Eating may constitute an intermediate step to ED (Duarte et al., 2016), the IEQ-G may serve as a screening instrument. Currently Duarte et al. (2016) favor the EDE-Q as a screening instrument for ED, but the IEQ assesses further aspects not covered by the EDE-Q, viz. the psychological aspect of rigidity in the context of eating. Thus, we consider the IEQ-G a valuable supplementary measure in ED screenings and assessments.

In the present study, Inflexible Eating as measured by the IEQ-G showed the expected associations to constructs, which may be associated to ED from a theoretical point of view. In fact, we found latent correlation coefficients sufficiently large to assume associations with both OCD and ED. This is in line with previous studies assessing the psychometric qualities of the IEQ (Duarte et al., 2016; Linardon, 2018; Tie et al., 2022). These correlation patterns may again indicate the potentially significant role of Inflexible Eating in the development of ED (e.g., AN) mentioned above. Longitudinal and experimental studies are required to establish potential causal links from Inflexible Eating to ED.

Regarding OCD or OC symptoms, we found a remarkably high correlation of ϕ = 0.41 with the IEQ, showing that such symptoms are involved in Inflexible Eating (which is in line with Mandelli et al., 2020 or Holland et al., 2014). This is not surprising, as both constructs share a common denominator: those affected feel uncomfortable when hindered at executing their rituals, which, if obeyed, provide them with a feeling of power and, if not, inferiority. In the case of Inflexible Eating (and ED), there is a lack of alignment, i.e., external stimuli, like food cues, and internal stimuli, like hunger, will not trigger food intake. Rather, it is dominated by self-imposed eating rules, i.e., rational behavior is overruled by impulses not related to nutrition. To this end, the present results support the findings of Simpson et al. (2013) in that OC symptoms should be addressed in ED assessment and the according therapeutic techniques considered as supplementary to ED treatment. Further research should, therefore, explore the potential of therapeutic interventions targeting OCD or OC symptoms in the context of ED.

Table 5 revealed that the non-omnivore respondents had larger correlations to the OC and the EDE-Q subscales than the entire sample. This might indicate that non-rational motives are associated with specific eating preferences. For instance, a respondent XX term “semi-vegetarian” (Timko et al., 2012) may pretend to just follow a certain diet but actually adhere to a disordered relationship with food. Heiss et al. (2018, 2020) and McLean et al. (2022b,c) also reported relationships of ED and specific dietary preferences. Interestingly, they found the EDE-Q performing sub-optimal in a vegan/vegetarian sample.

One finding, initially not formulated as a research question, was that the EDE-Q yielded rather mediocre psychometric properties. However, the majority of prior studies also reported inadequate model fit for the traditional 4-factor model and found various and inconsistent factor solutions regarding both, number of factor and items' allocation. Our findings were closest to those of Hilbert et al. (2007), who (also in a German speaking sample) favored the same 3-factorial solution. To our knowledge, none of the other studies exploring the psychometric properties of the EDE-Q mentioned the within-item-multidimensionality of item 8, which produced estimation problems in our analyses. Penelo et al. (2013), for instance, report estimation problems (non-positive definite matrix solution) for the original 4-factor model of the EDE-Q. It is quite plausible that item 8 and its within-item-multidimensionality caused their estimation problems as it did in our study. However, due to a lack of alternatives, we had to use this instrument as it still constitutes the most extensively validated ED assessment. Moreover, our decision to use the EDE-Q anyway is supported by the fact that the initial article introducing the concept of Inflexible Eating and the IEQ also used the EDE-Q as a reference (Duarte et al., 2017). Moreover, later studies referred to the EDE-Q as well (Linardon et al., 2019; Tie et al., 2022). The psychometric results of the IEQ excelled those of the EDE-Q. However, this might be due to the fact that the IEQ is much more focused on restraint eating with respect to both the rigid and the inflexible variants (cf. Westenhoefer, 1991; Duarte et al., 2017). In contrast, the EDE-Q captures a much broader concept involving more aspects of ED. Moreover, the EDE-Q addresses both intensity and frequency of various clinically important aspects (e.g., laxative abuse).

Both the IEQ and the EDE-Q are self assessments, which may prove difficult to apply in and ED or disordered eating population. Concealing tendencies may systematically bias the responses (not only regarding the BMI, see above; Vandereycken and Van Humbeeck, 2008), but also in the questionnaires' items themselves. The problems regarding the latent structure of the EDE-Q (Berg et al., 2012) may be due to such phenomena. However, early recognition has proven indispensable for avoiding manifestation of clinical ED, for which the IEQ may excel the EDE-Q for both, its psychometric advantages and psychological aspects of Dietary Restraint not covered by the EDE-Q. Moreover, the general problems associated to self-assessment in this population may be overcome by complementary techniques (cf. Smith et al., 2018; Elran-Barak et al., 2020).

In the present study it became apparent that on the one hand, ED of all kinds have specific peculiarities for males and females while, on the other hand, ED assessments (both IEQ and EDE-Q) ignore these differences entirely but rather reflect a feminine perspective (Mitchison and Mond, 2015), which can be traced back to the diagnostic criteria of DSM (American Psychiatric Association, 2013; although the amenorrhea criterion has been excluded from the latest edition) and ICD (World Health Organization, 2021). Thus, important diagnostic information may be overlooked, especially in males (Murray et al., 2017). More generally, not only the gender aspect is underrepresented but also peculiarities regarding age (Peat et al., 2008; Mulchandani et al., 2021), BMI, diet preferences (McLean et al., 2022a,c), and further specific populations. We found evidence for that claim in the latent correlation coefficients of the validity analysis which were 1) considerably higher in the female compared to the male subgroup, 2) considerably higher for the age group up to 34 compared to 35+, and 3) considerably higher for individuals with an BMI below 18.5. These findings may either show that these constructs are indeed more tightly related in females vs. males or that current ED assessment follows a too narrow concept. If the latter is the case, then it limits the validity of current instruments, which, in turn, might cause overseeing important groups also requiring psychological (or even medical) support. Therefore, we should think about instruments specifically designed for the specificities of either group.

We have, of course, to keep in mind that the present study is based on a convenience sample, hence a replication (possibly involving IRT models which are more flexible regarding sampling) is indicated. Moreover, the cross-sectional study design does not allow for assessing the instrument's stability. The current sample only allowed for analyzing respondents assigning themselves as male or female. Targeting specifically the LGBTQIA community would be both an interesting and important endeavor.

The German version of the IEQ presented in this article further has shown promising psychometric properties and seems applicable in a German population. Thus, it adds to cross-cultural assessment of ED or disordered or inflexible eating habits, which, if untreated, may develop into full ED (see Schaumberg et al., 2016 for an overview). Further research has to show, whether group specific norms are required and a more gender, age and diet sensitive extension might be thought of, as there is not one size that fits all.

## Data availability statement

The raw data supporting the conclusions of this article will be made available by the authors upon request, without undue reservation.

## Ethics statement

Ethical review and approval was not required for the study on human participants in accordance with the local legislation and institutional requirements. The patients/participants provided their written informed consent to participate in this study.

## Author contributions

AS, LM, and RWA designed the study, carried out the data collection, and wrote the manuscript. AS performed the analyses. RWA supervised the entire project and provided statistical expertise. All authors contributed to the article and approved the submitted version.
