# Supplementary material for: Strengths and weaknesses of the German translation of the Inflexible Eating Questionnaire and of eating disorder assessment in general
Source: Front Psychol. 2022 Dec 19;13:1002463. doi: 10.3389/fpsyg.2022.1002463 (PMC9806356; doi:10.3389/fpsyg.2022.1002463)
Supplement: Supplementary file 1 [file Table_1.pdf]

Supplementary Material

Strengths and weaknesses of the German translation of the Inflexible Eating Questionnaire and of eating disorder assessment in general

Anna Schultz, Linda Maurer, and Rainer W. Alexandrowicz

Table S1. Skewness and Kurtosis of the Items.

EDE-Q			SCL-90-R/O-C			IEQ-G		
Item	Skewness	Kurtosis	Item	Skewness	Kurtosis	Item	Skewness	Kurtosis
RS-01	0.96	−0.28	OC-01	1.15	0.45	IEQ-01	0.36	−1.16
RS-02	1.89	2.58	OC-02	1.55	2.15	IEQ-02	0.72	−0.55
RS-03	1.04	−0.06	OC-03	1.63	2.41	IEQ-03	0.23	−1.05
RS-04	1.18	0.07	OC-04	1.03	0.17	IEQ-04	0.15	−1.24
RS-05	2.56	5.95	OC-05	1.92	3.23	IEQ-05	0.31	−1.12
EC-07	2.82	8.18	OC-06	1.94	3.19	IEQ-06	0.87	−0.25
EC-09	2.63	6.51	OC-07	1.03	0.26	IEQ-07	0.87	−0.13
EC-19	3.35	12.85	OC-08	1.98	3.80	IEQ-08	0.89	−0.23
EC-20	1.79	2.81	OC-09	1.21	1.03	IEQ-09	0.04	−1.25
EC-21	3.04	9.40	OC-10	3.02	9.25	IEQ-10	0.99	−0.06
WCSC-08	2.82	7.96				IEQ-11	0.31	−1.20
WCSC-12	1.19	0.10						
WCSC-22	1.19	0.43						
WCSC-24	1.78	2.44						
WCSC-25	0.87	−0.30						
WCSC-06	0.80	−0.87						
WCSC-10	1.80	2.36						
WCSC-11	1.11	0.05						
WCSC-23	0.80	−0.45						
WCSC-26	0.69	−0.55						
WCSC-27	0.92	−0.26						
WCSC-28	0.98	−0.18						

Table S2. CFA loadings and Fit statistics EDE-Q: 2- and 4-factorial structure (Note: Standardized loadings and robust test statistics).

**Loadings**	**EDE-Q (2-factors)**				**EDE-Q (4-factors)**							
	Item	λ^*	Item	λ^*	Item	λ^*	Item	λ^*	Item	λ^*	Item	λ^*
	RS-01	.828***	C-08	.655***	RS-01	.824***	EC-07	.656***	WC-08	.645***	SC-06	.575***
	RS-02	.565***	C-12	.813***	RS-02	.568***	EC-09	.738***	WC-12	.801***	SC-10	.733***
	RS-03	.734***	C-22	.714***	RS-03	.731***	EC-19	.537***	WC-22	.699***	SC-11	.819***
	RS-04	.766***	C-25	.488***	RS-04	.765***	EC-20	.788***	WC-24	.477***	SC-23	.683***
	RS-05	.579***	C-25	.782***	RS-05	.588***	EC-21	.690***	WC-25	.779***	SC-26	.840***
			C-06	.586***							SC-27	.861***
			C-10	.774***							SC-28	.822***
			C-11	.825***								
			C-23	.689***								
			C-26	.796***								
			C-27	.814***								
			C-28	.785***								
			C-07	.566***								
			C-09	.653***								
			C-19	.438***								
			C-20	.743***								
			C-21	.577***								

**Fit**	**EDE-Q (2-factors)**	**EDE-Q (4-factors)**
χ^2 (df)	2452.29(208)***	2229.31(203)***
χ^2/df	11.790	10.981
CFI	0.763	0.790
TLI	0.737	0.761
RMSEA [CI]	0.130 [0.125-0.124]	0.124 [0.119-0.128]
SRMR	0.078	0.076
